# Role of SOCS2 in the Regulation of Immune Response and Development of the Experimental Autoimmune Encephalomyelitis

**DOI:** 10.1155/2019/1872593

**Published:** 2019-12-23

**Authors:** Allysson Cramer, Bruno Cabral de Lima Oliveira, Paulo Gaio Leite, David Henrique Rodrigues, Fatima Brant, Lisia Esper, Pollyana Maria Oliveira Pimentel, Rafael Machado Rezende, Milene Alvarenga Rachid, Antonio Lucio Teixeira, Ana Maria Caetano Faria, Mauro Martins Teixeira, Fabiana Simão Machado

**Affiliations:** ^1^Department of Biochemistry and Immunology, Institute of Biological Science, Federal University of Minas Gerais, Belo Horizonte, Brazil; ^2^Program in Health Sciences, Infectious Diseases and Tropical Medicine/Interdisciplinary Laboratory of Medical Investigation, Faculty of Medicine, Federal University of Minas Gerais, Belo Horizonte, Brazil; ^3^Ann Romney Center for Neurologic Diseases, Department of Neurology, Brigham and Women's Hospital, Harvard Medical School, Boston, MA, USA; ^4^Department of Pathology, Institute of Biological Science, Federal University of Minas Gerais, Belo Horizonte, Brazil

## Abstract

Multiple sclerosis (MS) is an inflammatory disease of the central nervous system (CNS). Experimental Autoimmune Encephalomyelitis (EAE) is the most widely used animal model for the study of MS. The Suppressor of Cytokine Signaling (SOCS) 2 protein plays a critical role in regulating the immune responses. The role of SOCS2 during EAE has not been explored. EAE was induced in WT and SOCS2^−/−^ mice using myelin oligodendrocyte glycoprotein (MOG_35-55_) peptide. Brain and spinal cord were examined during the peak (day 14) and recovery phase (day 28) of the disease. SOCS2 was upregulated in the brain of WT mice at the peak and recovery phase of EAE. The development of the acute phase was slower in onset in SOCS2^−/−^ mice and was associated with reduced number of Th1 (CD3^+^CD4^+^IFN-*γ*^+^) cells in the spinal cord and brain. However, while in WT mice, maximal clinical EAE score was followed by a progressive recovery; the SOCS2^−/−^ mice were unable to recover from locomotor impairment that occurred during the acute phase. There was a prolonged inflammatory response (increased Th1 and decreased Th2 and T regulatory cells) in the late phase of EAE in the CNS of SOCS2^−/−^ mice. Transplantation of bone marrow cells from SOCS2^−/−^ into irradiated WT mice resulted in higher lethality at the early phase of EAE. Altogether, these results suggest that SOCS2 plays a dual role in the immune response during EAE. It is necessary for damage during the acute phase damage but plays a beneficial role in the recovery stage of the disease.

## 1. Introduction

Multiple sclerosis (MS) is the most common demyelinating disease of the central nervous system (CNS). Patients can present motor deficits and sensory and sphincter problems, among other symptoms [1]. MS remains the main cause of neurological impairment in young adults after trauma [2].

Among many strategies to investigate the neuroinflammatory process of MS, a method commonly used to study MS *in vivo* is the induction of Experimental Autoimmune Encephalomyelitis (EAE) in mice. Animals with EAE present an autoimmune response against myelin antigens, a process that is also believed to be one of the hallmarks of MS [3]. The EAE model in mice induced by injection of myelin oligodendrocyte glycoprotein (MOG_35-55_) peptide is characterized by an early stage with myelin antigen presentation and reactive T cell expansion, and a late stage in which there is remission of disease signs. The neuroinflammatory process in EAE involves activation of microglia and macrophages, T cell infiltration in the CNS, and production of cytokines from T-helper (Th) 1 and Th17 cells [4]. This can result in demyelination, gliosis, and neurodegeneration that lead to clinical symptoms. Initially, the Th1 response profile, typified by cytokines such as IFN-*γ*, was thought to be the sole effector in this disease. However, further studies demonstrated that IL-17-secreting T cells also contribute to disease pathogenesis [5].

The Suppressor of Cytokine Signaling (SOCS) family proteins are essential regulators of cellular responses to cytokines and were initially thought to only downregulate specific cytokine signals and consequently to decrease proinflammatory signals [6]. SOCS proteins are rapidly induced upon enhanced cytokine levels, thereby creating a mechanism to regulate “undesirable” long-term cytokine responses [7]. To date, eight SOCS family genes (CIS and SOCS 1-7) have been reported [8]. SOCS protein family regulates Janus kinases/signal transducers and activator of transcription proteins (JAK/STAT) signaling, which are key regulators of cytokine signaling pathways. This regulation can occur through direct interaction or via the targeting of receptor of cytokine/JAK complex and other signaling proteins for proteasomal degradation [9, 10].

SOCS2 protein has been recognized to modulate the innate and adaptive immune response in different experimental models of infection, including models of *Toxoplasma gondii*, *Trypanosoma cruzi*, and *Plasmodium berghei* Anka infection. In these models, the participation of SOCS2 is complex and include the generation/differentiation of Th1, Th2, Th17, and T regulatory (Treg) cells [11–14]. Moreover, SOCS2 is critical to balance physiological functions of the brain, heart, and liver by controlling the production of neurotrophic factors, calcium handling, and prooxidative mechanisms [11, 12, 15]. The absence of SOCS2 enhanced Th2 differentiation, which contributed to the generation of type 2 allergic responses [15, 16].

SOCS2 is known to bind phosphorylated tyrosine residues on activated cytokine receptors and competitively or sterically impede binding sites; this is the suggested mechanism for the inhibition of STAT recruitment and activation. Thus, SOCS2, as well as SOCS1 and SOCS3, can influence the response to cytokines in T cell subsets through STAT pathway-mediated differentiation and function [17, 18]. Therefore, SOCS proteins may not only regulate negatively the cytokine expression but also the induction of Th1 responses.

Here, we show that SOCS2 is important for controlling the onset/peak and remission phases of EAE development via regulation of the T effector and T regulatory (Treg) cell infiltration in the CNS. Our work provides new insights into the signaling mechanisms involved in MS-induced CNS injury, paving the way for possible new therapeutic targets for this important clinical problem.

## 2. Material and Methods

### 2.1. Animals

Animal care and handling procedures were in accordance with the guidelines of the local animal ethics committee. Eight to ten-week-old female C57BL/6 (WT) were obtained from the main animal facility of the Universidade Federal de Minas Gerais (UFMG, Brazil). SOCS2^−/−^ mice were kindly provided by Dr. Warren S. Alexander (the Walter and Eliza Hall Institute of Medical Research, Australia). Animals were maintained in the animal facilities of the Laboratory of Immunoregulation, Department of Biochemistry and Immunology (UFMG, Brazil), with filtered water, food *ad libitum*, and in a controlled environment (temperature and humidity).

For generation of bone marrow chimeras, four to eight-week-old female mice were irradiated 9 Gy total-body radiation (source ^60^Co) in two doses at 2 h intervals to minimize gastrointestinal toxicity and then given an i.v. infusion of 1 × 10^7^ BM cells from C57BL/6 or SOCS2^−/−^ mice as previously described [19]. Split irradiation reduced the number of animals succumbing to irradiation to under 3%. Three to five mice per group were kept together in individually ventilated cages (IVC) and had ad libitum access to food and water. The experiments were performed 4–5 weeks after transplantation. The Animal Ethics Committee of UFMG approved all experimental procedures used (numbers 152/2012 and 253/2016).

### 2.2. EAE Induction and Daily Assessments of Disease in Mice

EAE was induced using an emulsion containing myelin oligodendrocyte glycoprotein (MOG_35-55_) peptide (MEVGWYRSPFSRVVHLYRNGK; NeoMPS), Complete Freund's Adjuvant (CFA), and 4 mg/mL attenuated *Mycobacterium tuberculosis* H37 RA (Difco Laboratories, Sparks, MD, USA) as described [20]. Briefly, each animal received 100 *μ*L of emulsion containing 100 *μ*g of MOG_35-55_. Pertussis toxin, (Sigma Chemical Co, St. Louis, MO, USA) 300 ng/animal, was injected intraperitoneally (i.p.) on the immunization day and 48 h later. Animals were evaluated daily using a clinical score scale. Disease severity was evaluated as follows: 0: no disease; 0.5: partial tail paralysis; 1: tail paralysis or waddling gait; 1.5: partial tail paralysis and waddling gait; 2: tail paralysis and waddling gait; 2.5: partial limb paralysis; 3: paralysis of one limb; 3.5: paralysis of one limb and partial paralysis of another; 4: complete hind limb paralysis; 4.5: complete hind limb paralysis and front limb weakness; and 5: death. Animals were also weighed at days 0, 3, 7, and daily after this.

### 2.3. Histology

At 14 and 28 days post EAE induction (dpi), mice were euthanized with ketamine/xylazine and brain and spinal cord removed. Tissues were immediately fixed in 4% buffered formalin, and the fragments were embedded in paraffin. Tissue sections (4 *μ*m thick) were stained with hematoxylin and eosin (H&E) and examined under light microscopy. Sections were captured with a digital camera (DEI-470; Optronics, Goleta, CA) connected to a microscope (IX70; Olympus, Center Valley, PA) with a magnification of ×200. Spinal cord pathology (inflammation) was graded from 0 to 4, considering severity of the lesions (none = 0, minimal = 1, mild = 2, moderate = 3, and marked = 4).

### 2.4. Western Blot

Whole cell extracts were obtained from the homogenized brain of control and immunized mice by using a lysis buffer (1% Triton X 100, 100 mM Tris/HCl, pH 8.0, 10% glycerol, 5 mM EDTA, 200 mM NaCl, 1 mM DTT, 1 mM PMSF, 25 mM NaF, 2.5 mg/mL leupeptin, 5 mg/mL aprotinin, and 1 mM sodium orthovanadate). Lysates were centrifuged at 13,000xg for 10 min at 4°C and quantified using the Bradford assay reagent from Bio-Rad (Hercules, CA). Protein extracts (80 *μ*g) were separated by electrophoresis on a denaturing 12% polyacrylamide-SDS gel and transferred onto nitrocellulose membranes. Membranes were blocked overnight at 4°C with PBS containing 5% (*w*/*v*) nonfat dry milk and 0.1% Tween-20, washed three times with PBS containing 0.1% Tween-20, and then incubated with anti-SOCS2 (1 : 1000, Cell Signaling). After washing, membranes were incubated with appropriate horseradish peroxidase-conjugated secondary antibody. Immunoreactive bands were visualized by using an enhanced chemiluminescence detection system (Amersham™ ECL™, GE Healthcare), as described by the manufacturer. The levels of SOCS2 were quantified by using a densitometric analysis software (ImageJ, Image Processing and Analysis in Java; NIH, Bethesda, MD), and the values were normalized to the constitutive *β*-actin levels in the same sample. Changes in protein levels were estimated, and the results were expressed as a SOCS2/*β*-actin ratio in arbitrary units.

### 2.5. Flow Cytometry

After euthanasia, the brain, spinal cord, and spleen were removed from mice, and leukocytes were isolated by homogenization into RPMI media. This cell suspension was fractionated using a step gradient consisting of 30% percoll (Sigma, St. Louis, MO) diluted in RPMI layered over 75% percoll diluted in RPMI. After centrifugation (8000xg), myelin was aspirated off the top of the 30% percoll layer. Leucocytes were removed from the interface between the 75% and the 30% layers of percoll. Afterwards, leucocytes were centrifuged (600xg) and resuspended in 1 mL of a solution containing 0.5% Bovine Serum Albumin (BSA), 2 mM azide, and phosphate-buffered saline (pH 7.4). Leukocytes obtained from the brain and spinal cord were stained with a combination of CD3 (APC-Cy7), CD4 (PE-Cy7), IL-10 (APC), CD25 (PerCP-Cy5), and FoxP3 (PE); CD3 (APC-Cy7), CD4 (PE-Cy7), and IFN-*γ* (APC); and IL-17A (PE). Data were acquired using BD FACSCanto II (Becton Dickinson, San José, CA, USA), and raw data of FACS analysis were processed using FlowJo software (Tree Star, Ashland, OR, USA).

### 2.6. Statistical Analysis

Results are shown as the mean ± SEM. Difference among groups was evaluated by using analysis of variance (ANOVA) followed by the Student-Newman-Keuls post hoc test or two-way ANOVA followed by the Bonferroni correction, as needed, for multiple comparisons when parametric assumptions were met. Otherwise, the Mann-Whitney *U* test was applied. GraphPad Prism Software (San Diego, CA, USA) was used to construct graphics. The level of significance was set as *p* < 0.05 or, in some data, *p* < 0.001.

## 3. Results

### 3.1. SOCS2 Regulates the Development of EAE

**Figure 1:**
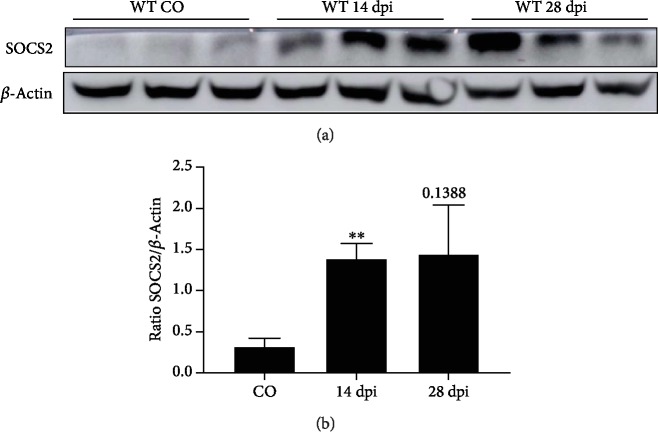
SOCS2 expression during EAE. WT and SOCS2^−/−^ mice received an injection of MOG_35-55_ subcutaneous to induction of EAE. At 14 and 28 days post injection (dpi), brain samples from WT and SOCS2^−/−^ mice were harvested and homogenized and Western Blotting analysis was performed using specific antibodies. (a) SOCS2 and *β*-actin expression. (b) Normalization graphic of the blots for SOCS2 is shown. *β*-Actin was used for normalization of protein load. Each band represents a different mouse. Data are representative of three independent experiments and shown as the mean ± SEM. ^∗∗^*p* = 0.01.

The involvement of SOCS2 on the development and/or protection during EAE is unknown. Initial experiments showed there was elevation of the expression of SOCS2 protein during the acute phase of EAE and found increased SOCS2 expression levels in the brains of mice at 14 and 28 days after EAE induction (Figures 1(a) and 1(b)).

**Figure 2:**
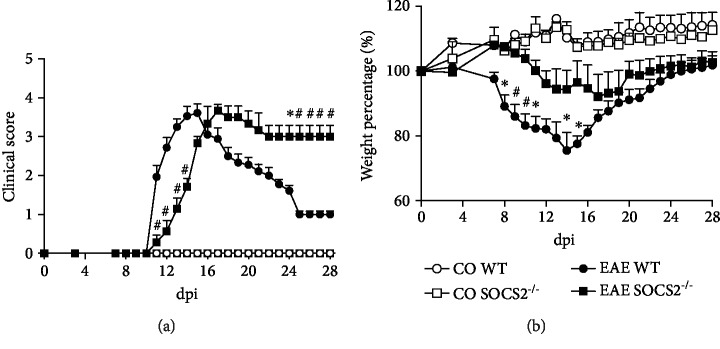
Regulatory role of SOCS2 during EAE. WT and SOCS2^−/−^ mice received an injection of MOG_35-55_ subcutaneous to induction of EAE. (a) Score was monitored by clinical signs comparing WT with SOCS2^−/−^ mice. (b) Weight loss percentage also was assessed daily comparing WT with SOCS2^−/−^ animals. Unimmunized WT and SOCS2^−/−^ mice were used as controls (*n* = 10 WT and 9 SOCS2^−/−^). Data represent the mean ± SEM. ^∗^*p* = 0.05; ^#^*p* = 0.01 (statistical significance was performed by Student's *T*-test or two-way ANOVA, with Tukey posttest).

Next, we characterized clinical signs of EAE in WT and SOCS2^−/−^ mice. Weight and clinical scores were evaluated at days 0, 3, 7, and then daily until 28 after induction of EAE. Motor impairment started at day 11 and peaked at day 14 in WT mice. Weight loss pattern followed changes in the clinical score (Figures 2(a) and 2(b)). Importantly, SOCS2-deficient mice showed strong resistance to EAE development, both at the onset and at the peak of the disease, with better motor function and control of body weight loss when compared with WT counterparts.

It is well known that WT animals subject to MOG-induced EAE develop a remission phase that follows the acute inflammatory injury of the CNS. As seen in Figure 2, after the peak of the disease, there was partial remission of locomotor dysfunction with a decrease in the clinical score and weight gain in WT (Figures 2(a) and 2(b)). However, SOCS2^−/−^ mice did not recover from CNS injury (Figure 2(a)), and the weight of these animals continued to decline until 19 dpi (Figure 2(b)). Thus, in the absence of SOCS2, disease onset and progression is delayed but recovery does not occur, suggesting that the expression of SOCS2 is detrimental at the onset/peak of EAE but benefic at the recovery stage of disease.

### 3.2. SOCS2 Modulates the Inflammation Level in the CNS during the Peak of EAE

**Figure 3:**
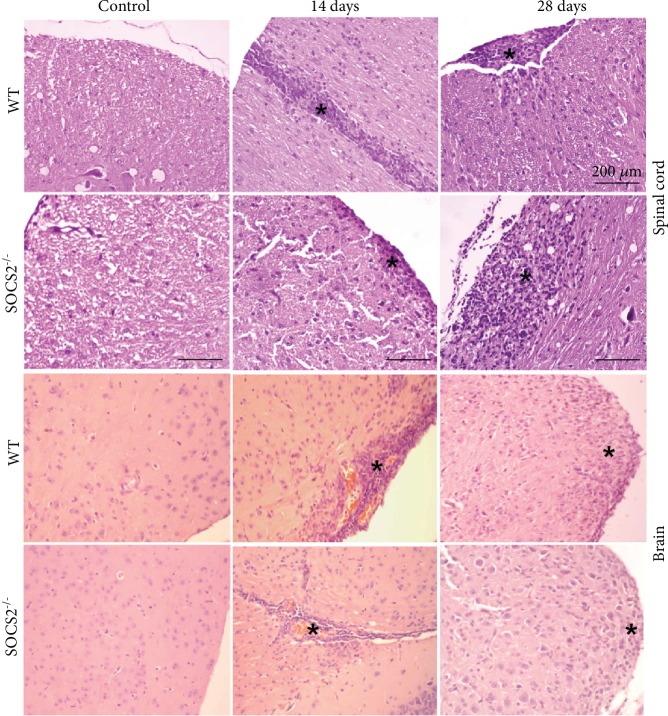
SOCS2 modulates inflammation in the CNS during EAE. Histology of brain (brainstem) and spinal cord sections from WT and SOCS2-deficient animals. Control mice with normal histological appearance. Brain, 14 days: EAE induced WT mice showing a locally extensive and intense infiltration of mononuclear cells (asterisk). EAE induced SOCS2^−/−^ mice with moderate perivascular inflammation (asterisk). Spinal cord, 14 days: EAE induced WT mice showing intense myelitis characterized by infiltration of mononuclear cells (asterisk). EAE induced SOCS2^−/−^ mice with mild to moderate myelitis (asterisk). Spinal cord at 28 days: EAE induced WT mice exhibiting mild to moderate meningomyelitis and EAE induced SOCS2^−/−^ mice showing locally extensive and intense meningomyelitis and demyelization (asterisks). Brain at 28 days: EAE induced WT mice exhibiting moderate encephalitis and induced SOCS2^−/−^ mice showing intense inflammatory infiltration (asterisks). Original magnification: ×200 (*n* = 6 animals per group). Data are representative of three independent experiments.

Since SOCS2 plays a crucial role in modulating inflammatory events in several tissues, we next investigated the inflammatory cell profile in the CNS of EAE-induced WT and SOCS2^−/−^ mice at 14 and 28 days after induction of EAE (Figure 3). Consistent with the clinical scores, WT mice showed pronounced infiltration of mononuclear cells in the neuropil, predominantly in the brainstem at 14 dpi (Figure 3). SOCS2^−/−^ mice exhibited a reduction in damage throughout the parenchyma with moderate inflammation at this time point. In the spinal cord, WT mice had severe myelitis characterized by intense cell infiltration at 14 dpi. Brain tissues from SOCS2^−/−^ mice showed a mild to moderate myelitis at this time point (Figure 3). At the peak of EAE, histological differences reflected directly in the clinical features. While WT mice had pronounced locomotor problems, with total tail paralysis and total or partial paralysis of the hind limbs in addition to urinary incontinence in some cases, SOCS2^−/−^ mice had significantly less locomotor problems (reduced paralysis), with less impairment in tail muscle tone and limb movement. Altogether, these data indicate that SOCS2 favors the inflammatory response during the early stage of EAE.

At 28 dpi, WT mice subject to MOG-induced EAE exhibited focal, mild to moderate meningomyelitis in the spinal cord (Figure 3). At this time point, extensive and intense meningomyelitis and demyelination were detected in SOCS2^−/−^ mice (Figure 3). SOCS2^−/−^ mice showed an increased inflammatory infiltration in the brain and spinal cord when compared with WT, which only exhibited moderate encephalitis (Figure 3).

### 3.3. SOCS2 Regulates T Cell Infiltration in the CNS during EAE

**Figure 4:**
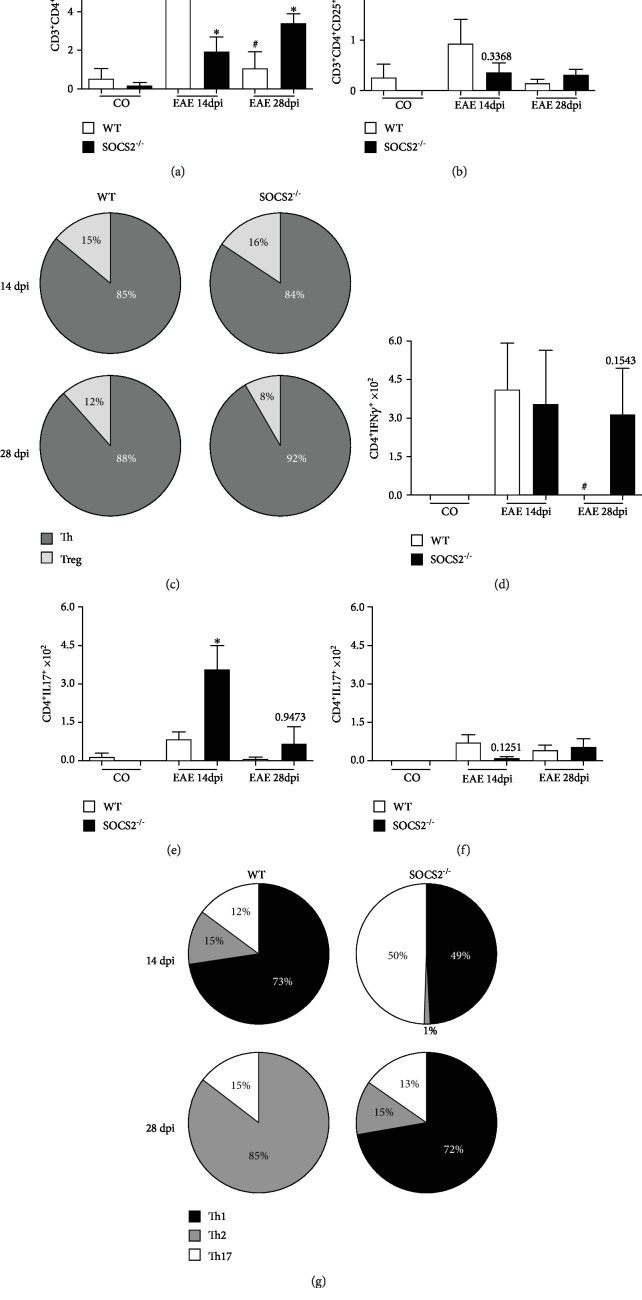
SOCS2 influences the infiltrating immune cell profile in the CNS. The spinal cord from WT and SOCS2^−/−^ naive or immunized (MOG) mice was harvested and submitted to Flow Cytometry analysis at 14 and 28 dpi. Numbers of CD3^+^CD4^+^ (a), CD4^+^CD25^hi+^FoxP3^+^ (b), CD4^+^CD3^+^IFN-*γ*^+^ (d), CD3^+^CD4^+^IL-17^+^ (e), and CD4^+^CD3^+^IL-10^+^ (f) of total population were assessed by Flow Cytometry using specific antibodies as described in Material and Methods. (c) Proportion of CD3^+^CD4^+^ (Th) and CD4^+^CD25^hi+^FoxP3^+^ (Treg). (g) Proportion of CD4^+^CD3^+^IFN-*γ*^+^ (Th1), CD3^+^CD4^+^IL-17^+^ (Th17), and CD4^+^CD3^+^IL-10^+^ (Th2) cells (*n* = 6 animals per group). Data are representative of three independent experiments and shown as the mean ± SEM. ^∗^*p* = 0.05, ^#^*p* = 0.05 (comparison between WT EAE 14 days and WT EAE 28 days).

A crucial event described during the onset and peak of EAE neuropathology is the infiltration of immune cells in the CNS [21]. We analyzed the infiltration of CD4 T cells, the primary subset involved in EAE development, in the spinal cord and brain from both WT and SOC2^−/−^ mice at 14 and 28 dpi. There was reduced number of T CD4 cells in the spinal cord from SOCS2^−/−^ mice at 14 dpi as compared with WT counterparts (Figure 4(a)). However, at the remission phase of EAE (28 dpi), SOCS2^−/−^ mice showed increased number of CD4 T cells (Figure 4(a)) and similar numbers of Treg cells as compared with WT mice in the spinal cord (Figure 4(b)). Importantly, the proportion of Th (CD3^+^CD4^+^) cells and Treg (CD4^+^CD25^hight^FoxP3^+^) cells in the spinal cord showed a reduction of Treg cells in SOCS2^−/−^ mice at 28 dpi as compared with WT mice (Figure 4(c)). We observed similar profile in brains (Figure [Supplementary-material supplementary-material-1]). Next, we investigated the profile of the infiltrated Th cells, including Th1, Th2, and Th17 cells in the spinal cord at the early and late phases of EAE. We found that the spinal cord from SOCS2^−/−^ mice showed increased numbers of Th17 cells and reduced numbers of Th1 cells at 14 dpi as compared with WT counterparts (Figures 4(e) and 4(f)). At the late phase of EAE, SOCS2^−/−^ mice showed a dramatic increase in Th1 numbers (Figure 4(d)). Moreover, the proportion of Th1 and Th2 cells was reduced at 14 dpi in SOCS2^−/−^ mice, whereas the proportion of Th17 cells was increased as compared with WT counterparts (Figure 4(g)). However, at 28 dpi, EAE-induced WT mice presented predominance of Th2 cells in the spinal cord, whereas SOCS2^−/−^ mice strongly increased Th1 cell frequency (Figure 4(g)).

### 3.4. Chimeric Mice Are Susceptible to EAE

**Figure 5:**
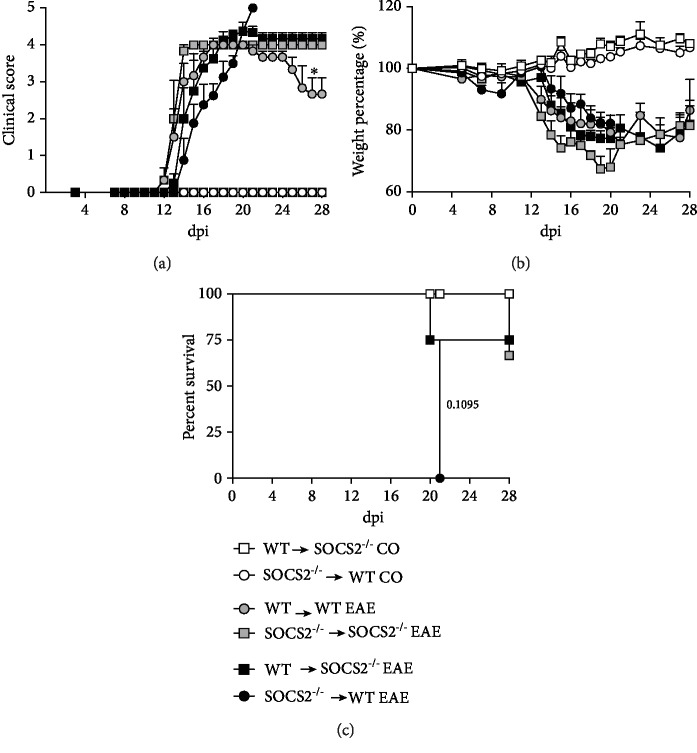
Role of SOCS2 in the hematopoietic and nonhematopoietic compartments in EAE development. EAE was induced in chimeric SOCS2^-/-donor^→WT EAE^host^, WT^donor^→SOCS2^−/−^ EAE^host^, WT^donor^→WT EAE^host^, and SOCS2^-/-donor^→SOCS2^−/−^ EAE^host^. (a) Score was monitored by clinical signs comparing WT with SOCS2^−/−^ mice. (b) Weight loss percentage and (c) survival were also assessed daily comparing WT with SOCS2^−/−^ animals. Data represent the mean ± SEM (statistical significance was performed by one-way ANOVA, with Tukey posttest and survival log rank test).

Thus far, our results demonstrated that SOCS2-deficient mice are resistant to the early phase of EAE but develop persistent disease at the late phase, an effect associated with increased infiltration of inflammatory cells into the CNS. To investigate whether nonhematopoietic cells were also involved in the dual effect of SOCS2 in EAE, we generated mice in which SOCS2 deficiency was restricted to either the nonhematopoietic or hematopoietic compartments. To address this, WT mice were irradiated and transplanted with bone marrow progenitor cells isolated from SOCS2^−/−^ donor (SOCS2^−/−^→WT). Conversely, irradiated SOC2^−/−^ mice were transplanted with WT bone marrow donor cells (WT→SOCS2^−/−^). Chimera mouse control was generated transferring WT or SOCS2^−/−^ bone marrow into irradiated WT or SOCS2^−/−^ mice, respectively (WT→WT and SOCS2^−/−^→SOCS2^−/−^). EAE was induced 4-5 weeks after transplantation. We found that all chimeric WT mice that received SOCS2^−/−^ cells (SOCS2^−/−^→WT), but not WT cells (WT→WT), died 20 days after EAE induction displaying parameters of severe disease (Figures 5(a)–5(c)). This suggests that SOCS2^−/−^ hematopoietic cells are extremely inflammatory and that SOCS2 expression in nonhematopoietic cells from WT mice cannot overcome inflammation. On the other hand, EAE in chimeric SOCS2^−/−^ mice that received WT or SOCS2^−/−^ cells was marked by a significant loss of the early resistance to the development of the clinical score displayed by EAE-induced SOCS2^−/−^ mice (Figure 5(a)). Moreover, despite 75% survival, SOCS2^−/−^ mice repopulated with WT or SOCS2^−/−^ cells were unable to recover at the late phase of disease (Figures 5(a)–5(c)), suggesting that SOCS2 in nonhematopoietic cells is important for the initial protection from EAE, whereas SOCS2 in hematopoietic cells is critical for mouse recovery from EAE. Thus, these results suggest that the deficiency of SOCS2 in either nonhematopoietic or hematopoietic compartments was sufficient to provide unbalanced inflammatory response that would result in disease-associated immunopathology.

## 4. Discussion

We describe here for the first time the role of SOCS2 in EAE. Our results can be summarized as follows: (i) SOCS2 is expressed in the CNS during EAE development; (ii) in the absence of SOCS2, clinical signs of severe EAE are delayed, and this delay is associated with reduced number of Th1 in the CNS; (iii) SOCS2^−/−^ mice eventually develop EAE but remission does not occur as in WT mice. Therefore, SOCS2^−/−^ mice are unable to recover from acute CNS damage. The lack of remission correlates with an uncontrolled immune response characterized by increased number of infiltrating Th1 inflammatory cells, and decreased number of Th2 and Treg cells in the CNS; (iv) not only SOCS2-deficient immune cells but also radioresistant or stromal cells deficient in SOCS2 result in higher susceptibility to EAE development. Together, our data suggest that SOCS2 contributes to the induction of neuroinflammation during the initial/peak phase of EAE and is also critical for recovery during the remission phase of the disease.

The inflammatory process in the CNS during EAE is associated with T cell infiltration, mainly Th1 and Th17 cells, and the participation of microglia and macrophages [22]. An essential event in EAE, which correlates with MS pathogenesis, is the restimulation of peripherally primed T cells that contribute to the amplification of the inflammatory response in the CNS. We showed that the deficiency of SOCS2 resulted in reduced proportion of Th1 cells infiltrating the brain and spinal cord at the peak of disease (day 14 post immunization). Together, fewer cells and cytokines may account for the reduction of inflammatory damage in the CNS. At the acute phase of EAE, IFN-*γ* and IL-17 production by infiltrating cells plays a crucial role in the inflammatory outcome. As Th1 cells are decreased in SOCS2^−/−^ mice, lower levels of IFN-*γ* may be associated with the initial and partial protection observed. These effects are likely to be reflected in subsequent events in the disease progression, since SOCS2^−/−^ mice showed a delay in EAE development as compared with WT mice. There are reports on the role of CCR6 in EAE development that the acute phase of the disease is associated with increasing frequencies of specific Th1 pathogenic cells and, at the same time, of Treg cells in the spinal cord of diseased mice [23]. These Treg cells were probably recruited to the inflamed tissue by CCR6-mediated chemotaxis and expanded there through the action of IL-2 produced by CD4^+^ effector cells on the CD25 (IL-2R*α* chain) that are highly expressed on Tregs [24]. However, we cannot rule out the involvement of SOCS2 in the regulation of signaling pathway downstream of CCR6 as well as of other chemokine receptors (or chemokine ligands) in the traffic of T cells into the CNS of EAE mice. Thus, a possible impairment in migratory mechanisms of leukocytes (both T cells and monocytes) may have contributed to the beneficial outcome observed during the initial phase of EAE in SOCS2^−/−^ mice.

Reduction in the inflammation found in SOCS2^−/−^ mice using this EAE model is consistent with results from our group that showed reduced inflammation in the brain of *P. berghei* Anka-infected mice at the early stage of the infection [11] and in the *T. cruzi*-infected heart of SOCS2^−/−^ mice [12]. Moreover, it has been shown that SOCS2 modulates/inhibits the inflammation in the CNS after *T. gondii* infection [14].

SOCS2 and some STAT family of transcription factor members, such as STAT5, are tightly correlated. For example, in the regulation of neuronal differentiation via Growth Hormone (GH), GH-activated STAT5b binds to the promoter of SOCS2 and leads to its expression, which in turn phosphorylates tyrosine residues on the GH receptor, resulting in reduced JAK2/STAT5 activation [25]. Because STAT5 activation is required for differentiation of Th1, Th2, and Treg (Foxp3^+^) cells and inhibition of Th17 polarization [26], we believe that this mechanism may also be involved in the delay of EAE development observed in SOCS2^−/−^ mice. However, deficiency of SOCS2 was reported to enhance Th2 differentiation in a murine model of allergy and asthma, in which STAT6 and STAT5 were activated, whereas STAT3, which is crucial for Th17 polarization was not [16]. We found that the absence of SOCS2 resulted in decreased number of Th2 at 14 and 28 dpi of EAE induction when compared with WT, but whether SOCS2 is essential for the Th2 differentiation during EAE still needs to be clarified. Of note, the proportion of Th2 cells in SOCS2^−/−^ mice was 5.6-fold lower than that of WT mice at 28 dpi, and its function does not appear to avoid/inhibit the Th1/Th17 responses. Therefore, both control of the recruitment of T cells and their polarization may underlie the effects of SOCS2 in EAE.

It has been previously demonstrated that the modulation of SOCS2 expression in dendritic cells is correlated with the expression of both 5-lipoxygenase (5-LO) and aryl hydrocarbon receptor (AhR) [14]. SOCS2 directly modulated 5-LO and AhR expression during the peak of EAE in the CNS (data not shown). Consistent with this, EAE-induced SOCS2^−/−^ mice presented a prominent reduction in inflammation in the CNS in the early phase of EAE. The Ah receptor is an important regulator of SOCS2 [14]. After stimulation, AhR is activated and translocated into the nucleus and can promote SOCS2 expression, leading to cytokine regulation [27]. AhR is activated by several ligands, including 5-LO products, such as lipoxin (LXA), described as crucial to induce SOCS2 in an AhR-dependent manner [14, 28]. Veldhoen et al. demonstrated that AhR modulates Th17 cell differentiation and then differentially regulates EAE, depending on the ligand type [29]. Since AhR^−/−^ mice are unable to recovery from the clinical signs after the peak of EAE has decreased the expression of SOCS2 and increased expression of NF-*κ*B in glial cells with subsequent inflammation and neurodegeneration in the spinal cord [30], our data suggest that the activity/expression of SOCS2 during the acute phase of EAE is probably a result of the AhR activation after 5-LO binding products. It is possible that when induced by a given stimulus, 5-LO produces LXA, which could activate AhR, leading to its translocation to the cellular nucleus followed by activation of *Socs2* gene. When expressed, SOCS2 protein would orchestrate the EAE outcome by regulating cytokine production, generation/expansion of protective and autoreactive immune cells, as well as by modulating the production of chemokines involved in the recruitment of more cells to the CNS during the acute phase of EAE. However, the production and role of eicosanoids during EAE need to be further investigated and are currently being tested in our laboratory.

The remission phase of EAE is characterized by partial clinical recovery with improved locomotor functions. Interestingly, SOCS2^−/−^ mice were unable to recover from the motor impairment after reaching a delayed peak of EAE. The reason for this incapacity to recover is not clear. Recent studies from our group have shown an unbalanced inflammatory response in the late stages of different models. For instance, in a model of liver failure with acetaminophen overdose, SOCS2^−/−^ mice had more necrosis, oxidation, and proinflammatory cytokines in the liver than WT mice [15]. In the cerebral malaria model, SOCS2^−/−^ mice display increased parasitemia and reduced Treg cell infiltration in the late phase, an effect associated with increased numbers of Th1 and Th17 cells and elevated cytokine levels [11]. Here, we demonstrated that SOCS2^−/−^ mice presented predominance of Th1 cell frequency in the late phase of the EAE. Interesting, other studies demonstrated that chronic inflammatory conditions in EAE caused a switch to alternative cytokines in Th17 cells, through generation and expansion of IFN-*γ*-producing Th17 cells [31, 32]. However, the role of SOCS2 in the plasticity of T cells during EAE development is unclear.

It has been shown that SOCS2 regulates inducible T regulatory cell (iTreg) stability via modulation of FoxP3 expression both *in vitro* and *in vivo*, and this subset is not stable after stimulation in the absence of SOCS2 protein [33]. In this context, Treg cells from SOCS2^−/−^ mice may not properly regulate the activity of effector T cells after stimulation. Consistent with this, we found that mice lacking SOCS2 showed significantly less proportion of infiltrated Treg cells in the CNS at the late stage of EAE. These events are probably associated with the uncontrolled activity of inflammatory T cells and higher inflammatory injury observed in the CNS of SOCS2^−/−^ mice in the late stages of EAE. Moreover, our chimeric mouse experiments clearly demonstrated that the deficiency of SOCS2 in either nonhematopoietic or hematopoietic compartments was sufficient to provide unbalanced inflammatory response that would result in disease-associated immunopathology. Thus, further studies are needed to determine the exact role of SOCS2 in the nonhematopoietic cells in the EAE context.

## 5. Conclusions

Altogether, our data suggest that SOCS2 is critical for recovery during the remission phase of EAE, probably by promoting Treg cell differentiation/expansion/stabilization with the subsequent dampening of proinflammatory cytokines. Thus, the SOCS2 protein is involved in the regulation of the inflammatory processes during EAE, and this mechanism may indicate an alternative target to current treatment strategies.

## Data Availability

The data used to support the findings of this study are available from the corresponding author upon request.

## Supplementary Materials

Supplementary Materials Supplementary 1: infiltrating immune cell profile in the SOCS2^−/−^ mouse brain. The brain from WT and SOCS2^−/−^ naive or immunized (MOG) mice were harvested and submitted to Flow Cytometry analysis at 14 and 28 dpi. Numbers of CD3^+^CD4^+^ (a) and CD4^+^CD25^hi+^FoxP3^+^ (b) of total population were assessed by Flow Cytometry using specific antibodies as describe in Material and Methods. (c) Proportion of CD3^+^CD4^+^ (Th) and CD4^+^CD25^hi+^FoxP3^+^ (Treg) in the brain. *n* = 6 animals per group. Data are representative of three independent experiments and shown as the mean ± SEM. ^∗∗^*p* = 0.01 (statistical significance was performed by Student's *T*-test).
