# Supplementary figures and images for: Role of SOCS2 in the Regulation of Immune Response and Development of the Experimental Autoimmune Encephalomyelitis
Source: Mediators Inflamm. 2019 Dec 23;2019:1872593. doi: 10.1155/2019/1872593 (PMC6942913; doi:10.1155/2019/1872593)

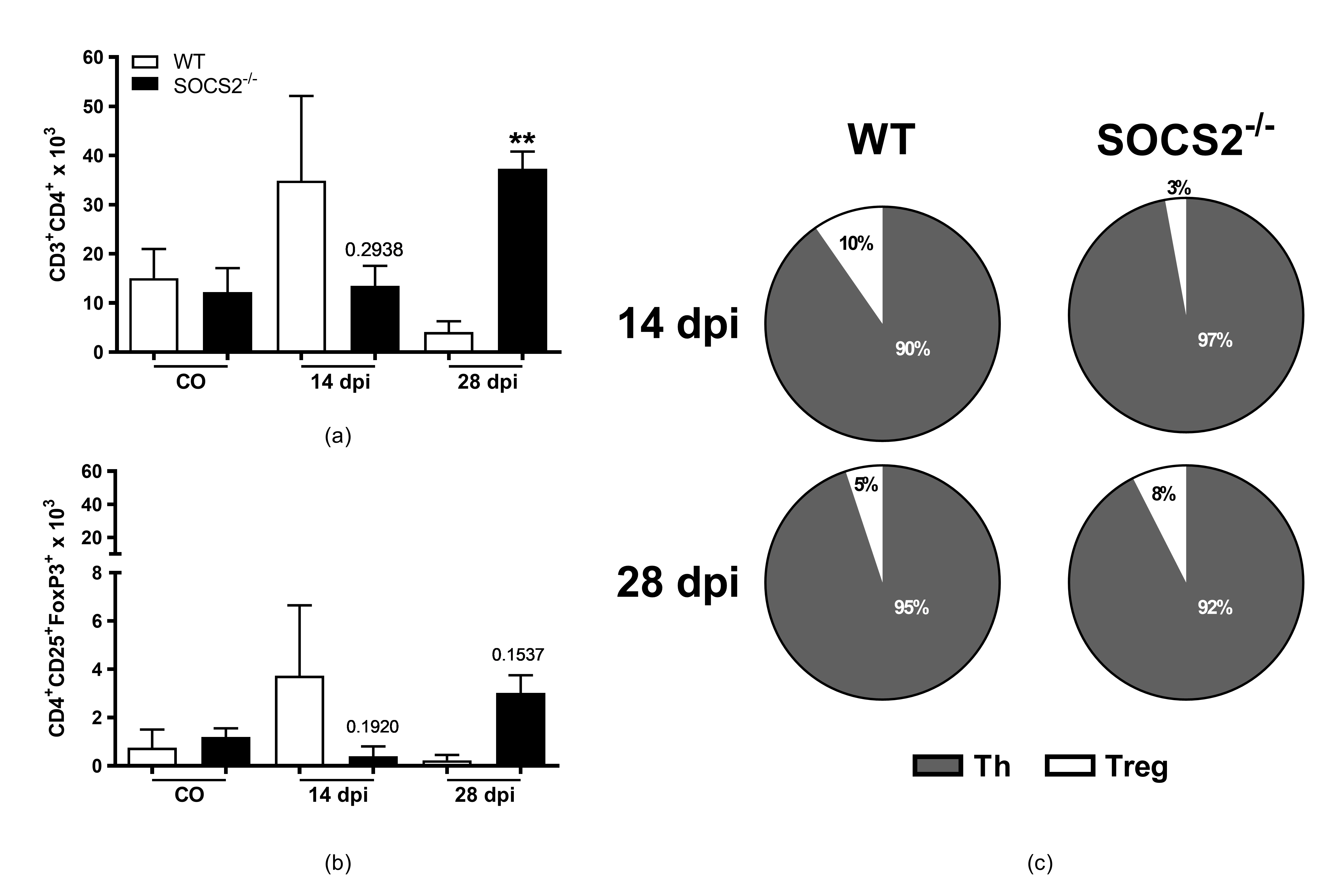

Supplement: Supplementary Materials — Supplementary 1: infiltrating immune cell profile in the SOCS2−/− mouse brain. The brain from WT and SOCS2−/− naive or immunized (MOG) mice were harvested and submitted to Flow Cytometry analysis at 14 and 28 dpi. Numbers of CD3+CD4+ (a) and CD4+CD25hi+FoxP3+ (b) of total population were assessed by Flow Cytometry using specific antibodies as describe in Material and Methods. (c) Proportion of CD3+CD4+ (Th) and CD4+CD25hi+FoxP3+ (Treg) in the brain. n = 6 animals per group. Data are representative of three independent experiments and shown as the mean ± SEM. ∗∗p = 0.01 (statistical significance was performed by Student's T-test). [file 1872593.f1.tif]
